# *T*-*Box20* inhibits osteogenic differentiation in adipose-derived human mesenchymal stem cells: the role of *T*-*Box20* on osteogenesis

**DOI:** 10.1186/s40709-019-0099-5

**Published:** 2019-09-18

**Authors:** Samaneh Mollazadeh, Bibi Sedigheh Fazly Bazzaz, Vajiheh Neshati, Antoine A. F. de Vries, Hojjat Naderi-Meshkin, Majid Mojarad, Zeinab Neshati, Mohammad Amin Kerachian

**Affiliations:** 1 Natural Products and Medicinal Plants Research Center, North Khorasan University of Medical Sciences, Bojnurd, Iran; 2 Biotechnology Research Center, Pharmaceutical Technology Institute, Mashhad University of Medical Sciences, Mashhad, Iran; 3 Department of Food and Drug Control, School of Pharmacy, Mashhad University of Medical Sciences, Mashhad, Iran; 4 School of Pharmacy, Mashhad University of Medical Sciences, Mashhad, Iran; 5 Department of Cardiology, Leiden University Medical Center, Leiden, The Netherlands; 6 Stem Cell and Regenerative Medicine Research Group, Academic Center for Education, Culture Research (ACECR), Khorasan Razavi Branch, Mashhad, Iran; 7 Medical Genetics Research Center, Mashhad University of Medical Sciences, Mashhad, Iran; 8 Department of Medical Genetics, Faculty of Medicine, Mashhad University of Medical Sciences, Mashhad, Iran; 9 Department of Biology, Faculty of Science, Ferdowsi University of Mashhad, Mashhad, Iran

**Keywords:** Adipose tissue-derived mesenchymal stem cells, Lentiviral vector, Osteogenesis, Transcription factor, *Tbx20*

## Abstract

**Background:**

Skeletal development and its cellular function are regulated by various transcription factors. The T-box (Tbx) family of transcription factors have critical roles in cellular differentiation as well as heart and limbs organogenesis. These factors possess activator and/or repressor domains to modify the expression of target genes. Despite the obvious effects of *Tbx20* on heart development, its impact on bone development is still unknown.

**Methods:**

To investigate the consequence by forced *Tbx20* expression in the osteogenic differentiation of human mesenchymal stem cells derived from adipose tissue (Ad-MSCs), these cells were transduced with a bicistronic lentiviral vector encoding *Tbx20* and an enhanced green fluorescent protein.

**Results:**

*Tbx20* gene delivery system suppressed the osteogenic differentiation of Ad-MSCs, as indicated by reduction in alkaline phosphatase activity and Alizarin Red S staining. Consistently, reverse transcription-polymerase chain reaction analyses showed that *Tbx20* gain-of-function reduced the expression levels of osteoblast marker genes in osteo-inductive Ad-MSCs cultures. Accordingly, *Tbx20* negatively affected osteogenesis through modulating expression of key factors involved in this process.

**Conclusion:**

The present study suggests that *Tbx20* could inhibit osteogenic differentiation in adipose-derived human mesenchymal stem cells.

## Introduction

Bone is a dynamic tissue undergoing continuous remodeling, which is coordinately regulated by osteoblasts and osteoclasts [1]. A proper balance between bone formation by osteoblasts and bone resorption through osteoclasts guaranties bone maintenance, whereas an imbalance in the activity of these cells leads to bone-associated problems including osteoporosis, osteopetrosis, Padget’s disease of the bone and rheumatoid arthritis [1–3]. Although the transcriptional regulatory networks involved in bone formation are rather well-defined, the discovery of new transcription factors involved in osteogenesis could further advance our understanding of normal and pathological bone development and homeostasis [4].

The T-box (Tbx) family of transcription factors has widespread roles in development and response pathways [5, 6]. T-box proteins contain a highly conserved DNA-binding domain of 180–200 amino acids, so-called T-box domain [4, 5, 7]. Based on the amino acid sequence of the T-box domain, Tbx family members are divided into five subfamilies: T, Tbx1, Tbx2, Tbx6, and Tbr1 [8]. Together with *Tbx1*, *Tbx10*, *Tbx15*, *Tbx18* and *Tbx22*, *Tbx20* belongs to the Tbx1 subfamily. T-box genes are involved in the development of the heart, limbs, mammary glands and cancers [4, 5]. Since T-box is highly conserved in T-box family [9], the members of this family may have similar DNA-binding properties. It has been demonstrated that *Tbx2*, *Tbx3*, *Tbx4*, and *Tbx5* are extensively detectable in the limb buds and their effects on embryonic skeletogenesis have been well-documented [10, 11]. T-box transcription factors contain activator, repressor or possess both domains, which together with other transcription factors affect the transcriptional activity of target genes in a cell type-specific manner [8, 12]. Studies have shown that *Tbx20*a, i.e. the dominant *Tbx20* isoform in the heart, can act both as a transcriptional activator and repressor. *Tbx20* is required for vertebrate cardiogenesis [13, 14], as revealed by proliferation and chamber and valve formation [7]. Besides, it has been shown that *Tbx20* is expressed in different tissues including heart, eye, ventral neural tube and limbs, thus it controls their developmental process [15]. A recent DNA microarray study suggests that skeletal development and cardiac valve maturation are accompanied with changes in the expression of overlapping sets of transcription factor genes including *Twist1*, *Sox9*, *Msx1*, and *Msx2* [16]. Recently, it was also reported that *Tbx20* plays a pivotal role in facial development [17]. Other T-box genes (e.g. *Tbx2*, *Tbx3*, *Tbx18* and *Tbx22*) are involved in bone formation as well as cardiac development [5, 18, 19]. Although the impact of *Tbx20* on heart development is well-investigated [14] its role in bone development has not been studied. To take into account the potential role of *Tbx20* in bone progenitors, we hypothesized that *Tbx20* may be an important modulator of osteoblast function. To test this idea, *Tbx20* was overexpressed in adipose-derived human mesenchymal stem cells (Ad-MSCs) using a vesicular stomatitis virus G protein-pseudotyped human immunodeficiency virus type 1 (HIV1)-based lentiviral vector. These vectors are extensively used in experimental and clinical studies because of their broad tropism, high efficiency and ability to mediate stable transgene in dividing and non-dividing cell populations [20].

## Materials and methods

### Cell culture and markers

Ad-MSCs were obtained from healthy individuals with the approval of the Ethics Committee of Mashhad University of Medical Sciences and with written informed consent of the donors. Ad-MSCs were extracted and cultured by a previously published protocol [21, 22]. Briefly, Ad-MSCs from lipoaspirate wastes of healthy donors undergoing aesthetic surgery were isolated by digestion with collagenase type I (0.075%) (Invitrogen, Massachusetts, USA) for 45 min at 37 °C in a shaker water bath followed by the addition of Dulbecco’s modified Eagle’s medium-low glucose (DMEM-LG) (Gibco BRL, Paisley, Scotland) supplemented with 20% fetal bovine serum (FBS; Gibco BRL, Paisley, Scotland) to inactivate the enzyme. The resulting cell suspension was subsequently centrifuged at 1000*g* for 15 min, and the cell pellet was then suspended in DMEM-LG containing 15% FBS, and 1× penicillin/streptomycin (Pen/Strep; Gibco). The cell suspension was maintained at 37 °C and 5% CO_2_ in T75 culture flasks. After 3 days, the cells were washed with phosphate-buffered saline (PBS) and cultured in fresh medium, which was subsequently replaced twice a week. The identity of the Ad-MSCs was verified by flow cytometry (BD FACSCalibur System, Biosciences, San Jose, CA) following immunostaining for cell surface antigens at passage three. Mouse monoclonal antibodies directed against CD11b, CD34, CD44, CD45, CD90, and CD105 were purchased from Acris Antibodies (Herford, Germany). FITC-conjugated goat anti-mouse antibodies were obtained from Biolegend (San Diego, CA).

To induce osteogenic differentiation, cells were kept in basal medium containing 0.1 µM dexamethasone, 50 μg/ml ascorbic acid and 10 mM β-glycerophosphate (all from Sigma-Aldrich Chemie, Taufkirchen, Germany). Adipogenesis was induced in the same medium but with 100 µM indomethacin (Sigma-Aldrich Chemie, Taufkirchen, Germany) instead of the ascorbic acid.

### Plasmid construction

DNA modifying enzymes used in this experiment were purchased from New England Biolabs (Bioke, Leiden, the Netherlands) and Fermentas (Fisher Scientific, Fermentas, the Netherlands) and applied as specified or by using standard protocols.

**Fig. 1:**
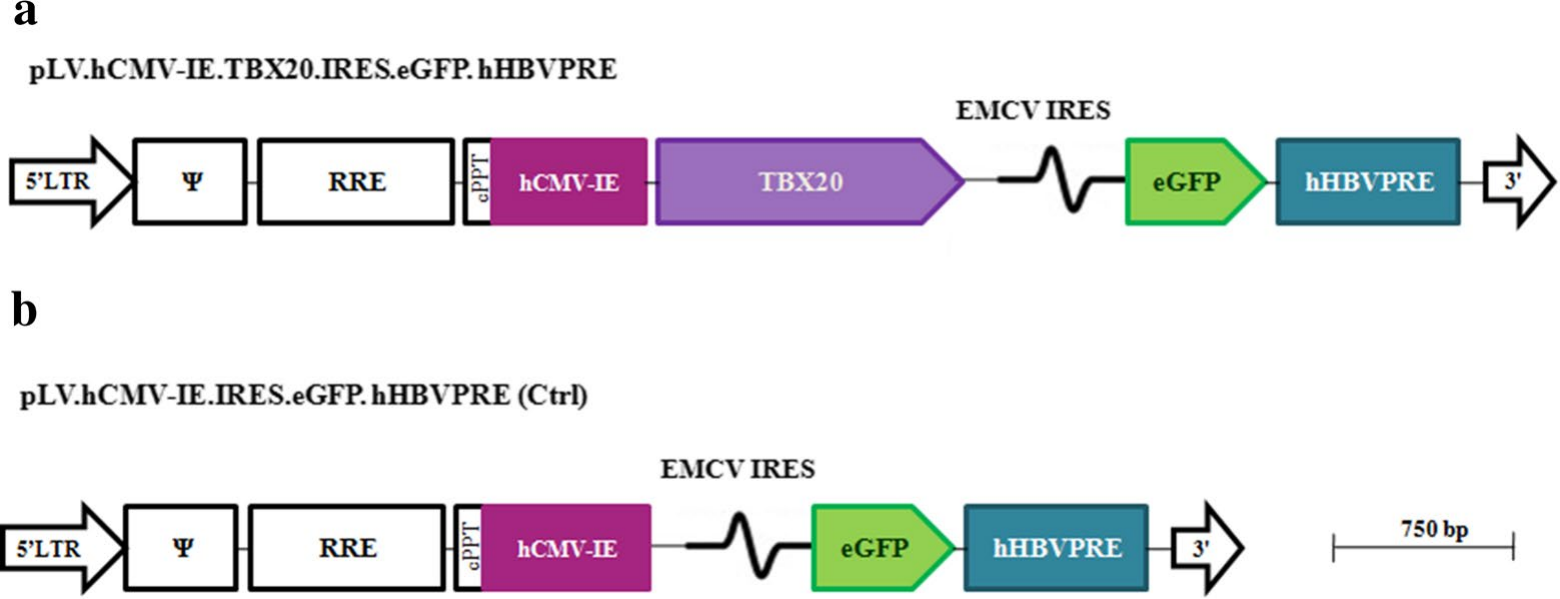
Map of LV DNA in the LV shuttle plasmids. pLV.Tbx20 (**a**), pLV.Ctrl (**b**). *5′ LTR* chimeric 5′ long terminal repeat including the Rous sarcoma virus U3 region as well as the HIV1 R and U5 regions. *Ψ* HIV1 packaging signal, *RRE* HIV1 Rev-responsive element, *cPPT* HIV1 central polypurine tract and termination site, *hCMV-IE* human cytomegalovirus immediate early gene promoter, *Tbx20* coding sequence of desired gene, *EMCV IRES* encephalomyocarditis virus internal ribosomal entry site, *EGFP* enhanced green fluorescent coding sequence, *hHBVPRE* human hepatitis B virus posttranscriptional regulatory element, *3′ LTR* 3′ HIV1 long terminal repeat involving a deletion in the U3 region to render the LV self-inactivating

The murine *Tbx20*-coding sequence was excised from plasmid pCRII Topo-*Tbx20* (Addgene, Cambridge, MA; plasmid number: 24715) with *Eco*RV and *Spe*I and inserted into the polylinker of the lentiviral vector shuttle construct pLV.hCMV-IE.IRES.EGFP.hHBVPRE that was previously known as pLV-CMV-IRES-EGFP [23] digested with the same enzymes. The resulting plasmid, which codes for *Tbx20* and the enhanced green fluorescent protein (EGFP), was designated pLV-*Tbx20*. To verify the correctness of pLV-*Tbx20*, restriction endonuclease digestions and nucleotide sequence analysis were performed. Sequencing was carried out by Leiden Genome Technology Center (http://www.lgtc.nl/) using a BigDye Terminator v3.1 Cycle Sequencing Kit and a 3730xl DNA Analyzer (both from Applied Biosystems). A schematic genetic maps of pLV-*Tbx20* and pLV-Ctrl constructs are provided in Fig. 1.

### Lentiviral vector production

To produce lentiviral vector particles, human embryonic kidney (HEK) 293T cells were co-transfected at a molar ratio of 1:1:2 with packaging plasmids psPAX2 (Addgene; plasmid number; 12260) and pLP/VSVG (Life Technologies Europe) and with lentiviral vector shuttle plasmid pLV-*Tbx20* (to generate LV-*Tbx20* particles) or pLV.hCMV-IE.IRES.EGFP.hHBVPRE (to produce control vector [LV-Ctrl] particles). Human embryonic kidney 293T cells were grown in DMEM-HG supplemented with 10% FBS. After the cells reached 70–80% confluency, the transfection medium was added containing the packaging plasmids supplemented with NaCl 150 mM and 25 kDa linear polyethylenimine (PEI; Polysciences Europe, Hirschberg an der Bergstrasse, Germany). The DNA–PEI complexes were left on the cells overnight. The next day, the transfection medium was replaced by DMEM-LG supplemented with 5% FBS and 25 mM HEPES-NaOH (pH 7.4). Producer cell supernatants were collected 48 h after the start of the transfection procedure and following low speed centrifuge, they were filtered through 0.45-μm pore-sized, 33-mm diameter polyethersulfone Millex-HP syringe filters (Millipore, Amsterdam, The Netherlands). To concentrate the vector particles, the cleared supernatants were underlayed with a cushion of 20% (wt/vol) sucrose followed by ultracentrifugation at 15,000 revolutions per minute and 4 °C for 2 h in a SW32 swinging bucket rotor (Beckman Coulter Nederland, Woerden, The Netherlands). The supernatant was removed completely and viral vectors were then suspended in PBS containing 1% bovine serum albumin (Sigma-Aldrich Chemie, Taufkirchen, Germany). Finally, viral vectors were aliquoted in 1.5 ml microtubes and stocked at − 80 °C for subsequent investigations.

### Lentiviral transduction

Ad-MSCs were plated at density of 5 × 10^3^ cells/cm^2^. The next day, the cells were infected with LV-*Tbx20* or LV-Ctrl particles in culture medium containing 2 µg/ml DEAE-dextran sulfate (Sigma-Aldrich Chemie, Taufkirchen, Germany) under antibiotic-free conditions. After 24 h, the cells were washed with PBS and the transfection medium was replaced with osteogenic differentiation medium. The transduction efficiency was checked by direct EGFP fluorescence microscopy. All experiments were performed in triplicate.

### Mineralization and alkaline phosphatase (ALP) activity assays

To stain calcific deposits as previously described [24], after 4th week of differentiation, cells were fixed with 10% formalin (Sigma-Aldrich Chemie, Taufkirchen, Germany) for 45 min at room temperature (RT). After washing away the fixative with water, the fixed cells were incubated with Alizarin Red S (ARS, Sigma-Aldrich Chemie, Taufkirchen, Germany) staining solution of pH 4.1–4.3 for 45 min at RT. Next, unbound ARS was aspirated out and washed away with water. Then, mineralized/red bone nodules were visualized by light microscopy (Nikon Eclipse TE2000-U, Tokyo, Japan). Stained cells were subjected to ARS quantification using 10% acetic acid (Sigma-Aldrich Chemie, Taufkirchen, Germany). The resulting slurry was transferred to microcentrifuge tube followed by heating at 85 °C and cooling on ice. Then, the tubes were centrifuged at 20,000*g* for 15 min and 10% ammonium hydroxide was added to the supernatant. In the final step, absorbance was read at 405 nm using a BioTek plate reader (Bad Friedrichshall, Germany). To detect alkaline phosphatase (ALP) activity, cells were cultured in differentiation medium for about 21 days. Then, they were fixed with 10% formalin, washed with PBS, and finally stained with BCIP/NBT (5-bromo-4-chloro-3-indolyl phosphate/nitroblue tetrazolium; Sigma, Germany). The ALP activity was visualized as a dark purple color. To measure ALP activity, differentiated cells were detached with lysis buffer (50 mM Tris–HCl [pH 7.4]), 1% Triton X-100 (Merck, Germany) followed by incubating with *p*-nitro-phenyl phosphate (pNPP; Sigma-Aldrich Chemie, Taufkirchen, Germany) for 30 min at RT. Finally, the chromogenic reaction product (i.e.; *p*-nitro-phenol [pNP]) was detected spectrophotometrically at 405 nm.

### Immunostaining

Two weeks after osteogenic induction, cells were fixed with 4% paraformaldehyde (in 1× PBS) at 4 °C for 30 min, permeabilized by incubation with 0.1% Triton X-100 in PBS and incubated overnight at 4 °C with mouse anti-alkaline phosphatase (ALP; BD Biosciences, San Diego, CA) and anti-collagen type I (ColI; Sigma-Aldrich Chemie, Taufkirchen, Germany), diluted as 1:200 and 1:1000 in PBS and 0.1% donkey serum, respectively (Santa Cruz Biotechnology, Santa Cruz, CA). The next day, cells were incubated at RT for 4 h with Alexa Fluor 568-conjugated donkey anti-mouse IgG (H + L) secondary antibody (Life Technologies Europe, Bleiswijk, The Netherlands) diluted as 1:400 in PBS and 0.1% donkey serum. Then, nuclei were incubated with Hoechst 33258 (Sigma-Aldrich Chemie, Taufkirchen, Germany) for 10 min at RT. After each incubation step, cells were thoroughly rinsed with PBS. Pictures were taken with an inverse fluorescence microscope (Nikon eclipse TE2000-U) attached to digital color camera. ImageJ, version 4.1 (National Institutes of Health, Bethesda, MA) was used for mean fluorescence signal intensity.

### Reverse transcription-quantitative polymerase chain reaction (RT-qPCR) analyses

To measure transcript levels of various genes, total RNA was isolated from cells at 7 and 14 days of osteogenic differentiation using Hybrid-R™ RNA purification kit (GeneAll, Seoul, Republic of Korea). Next, first strand-cDNA was synthesized by reverse transcription with GeneAll’s HyperScript kit (Seoul, Republic of Korea). Expression levels of desired genes was accessed using SYBR Green (Amplicon, Odense, Denmark)-based real time PCR (StepOne detection system; Applied Biosystems, CA, USA). Glyceraldehyde 3-phosphate dehydrogenase (*GAPDH*) was applied as reference gene and gene expression changes were calculated using 2^−ΔΔCt^ method. Forward and reverse primer sequences (Takapouzist, Tehran, Iran) used are shown in Table 1.

**Table 1:** RT-qPCR primers

Symbol	Name	Primer
ALPL	Alkaline phosphatase	F: 5′-CATGCTGAGTGACACAGACAAGAAG-3′ R: 5′-TGGTAGTTGTTGTGAGCATAGTCCA-3′
COL1A1	Collagen type I alpha 1	F: 5′-CATCTCCCCTTCGTTTTTGA-3′ R: 5′-CCAAATCCGATGTTTCTGCT-3′
RUNX2	Runt-related transcription factor 2	F: 5′-GGAGTGGACGAGGCAAGAGTTT-3′ R: 5′-AGCTTCTGTCTGTGCCTTCTGG-3′
OPN	Osteopontin	F: 5′-ATGATGGCCGAGGTGATAGT-3′ R: 5′-ACCATTCAACTCCTCGCTTT-3′
Tbx20 (human)	Homo sapiens T-box 20	F: 5′-CAAGCCCCAACTCTCCTCTC-3′ R: 5′-CTCCACCAAACTCCCCATGA-3′
Tbx20 (murine)	Murine T-box20	F: 5′-ATACCGCTATGCCTACCACC-3′ R: 5′-TATGGCCGTGTTGATCCAGT-3′
GAPDH	Glyceraldehyde-3-phosphate dehydrogenase	F: 5′-ATGTTCGTCATGGGTGTGAAC-3′ R: 5′-CACAGTCTTCTGGGTGGCAG-3′

### Statistical analysis

All experiments were performed in triplicate and all data were expressed as the mean ± standard deviation (SD). The statistical analysis was performed using Prism 6 (Graphpad Software, San Diego, CA). Significant associations between various groups were analyzed by ANOVA and defined as *p* values.

## Results

### Identification of human Ad-MSCs

The mesenchymal stem cells isolated from human adipose tissue had a spindle-shaped fibroblastic appearance and could form single-cell. As previously demonstrated, immunostaining and flow cytometric analysis revealed that Ad-MSCs expressed the mesenchymal stem cell markers CD44, CD90, CD105 at their surface while they were negative for CD11b, CD45, CD34 [21]. Culturing of Ad-MSCs in osteogenic differentiation medium stimulated calcification as evidenced by the bright-red mineralized nodules observed following ARS staining. Moreover, when the Ad-MSCs were cultured in adipogenic differentiation medium, the cells accumulated lipid droplets as indicated by Oil Red O. The results of the osteogenesis and adipogenesis assays showed that the Ad-MSCs possess multilineage differentiation potential capacities (Additional file 1: Fig. S1A and B).

### Production and functional testing of LV-*Tbx20* and LV-Ctrl particles

Twenty-four hours after the addition of the lentiviral vector shuttle and packaging plasmids, essentially all 293T producer cells were EGFP positive indicative of a high transfection efficiency. To test the gene transfer activity of the LV-*Tbx20* and LV-Ctrl preparations, 5 × 10^4^/cm^2^ 293T indicator cells were incubated with 2 μl of a 1000-fold dilution of both vector stocks. This resulted in the expression of EGFP in virtually all cells confirming that both vectors were functional and had a similar titer (Additional file 2: Fig. S2A, B).

### The effect of *Tbx20* overexpression on the osteogenic differentiation ability of Ad-MSCs

**Fig. 2:**
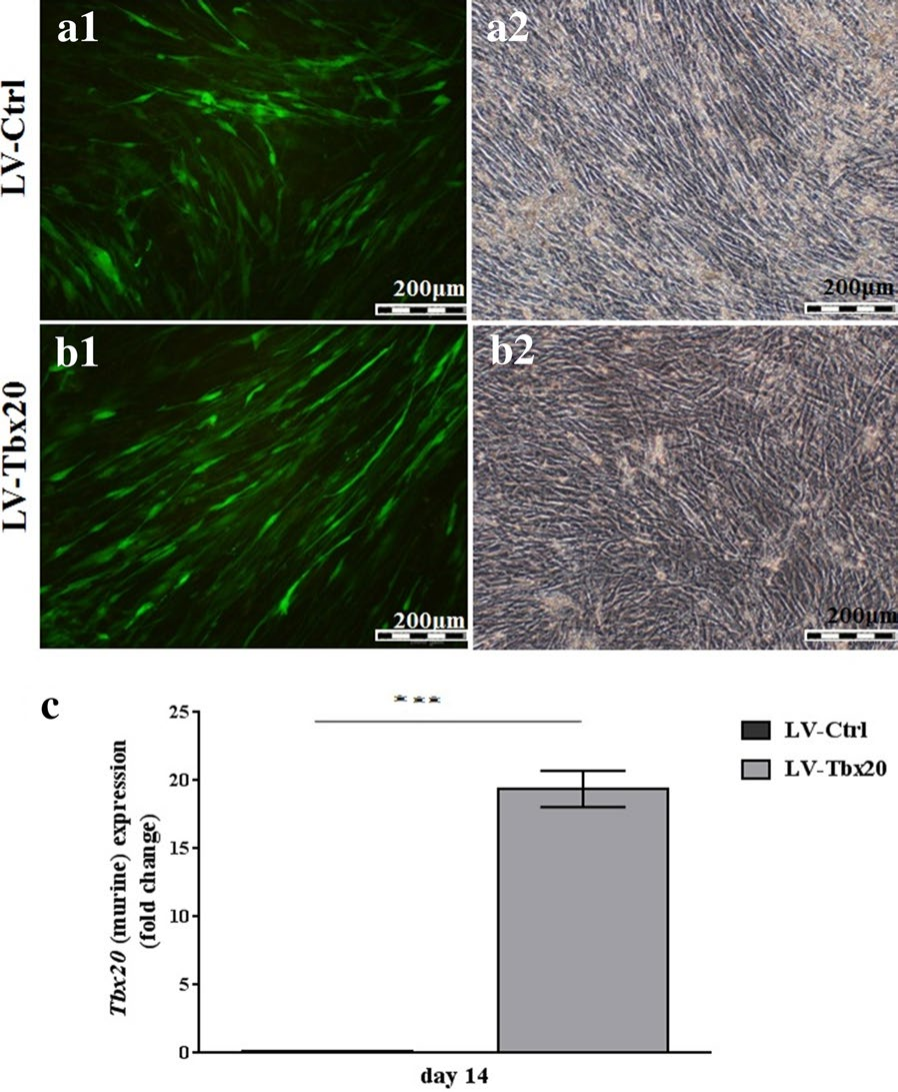
Fluoromicrographs (**a1**, **a2**) and brightfield images (**b1**, **b2**) of LV-Ctrl (**a1**, **b1**)- or LV-*Tbx20* (**a2**, **b2**)-transduced Ad-MSCs that were maintained in osteogenic differentiation medium for 21 days. **c** Expression of *Tbx20* (murine) in transduced cells, 14 days after transduction. RT-qPCR demonstrated that the *Tbx20* expression in LV-*Tbx20* transduced cells was around 20 times higher than its expression in LV-Ctrl transduced cells.***Indicates significant differences (*p *< 0.001) between LV-Ctrl and LV-*Tbx20*

**Fig. 3:**
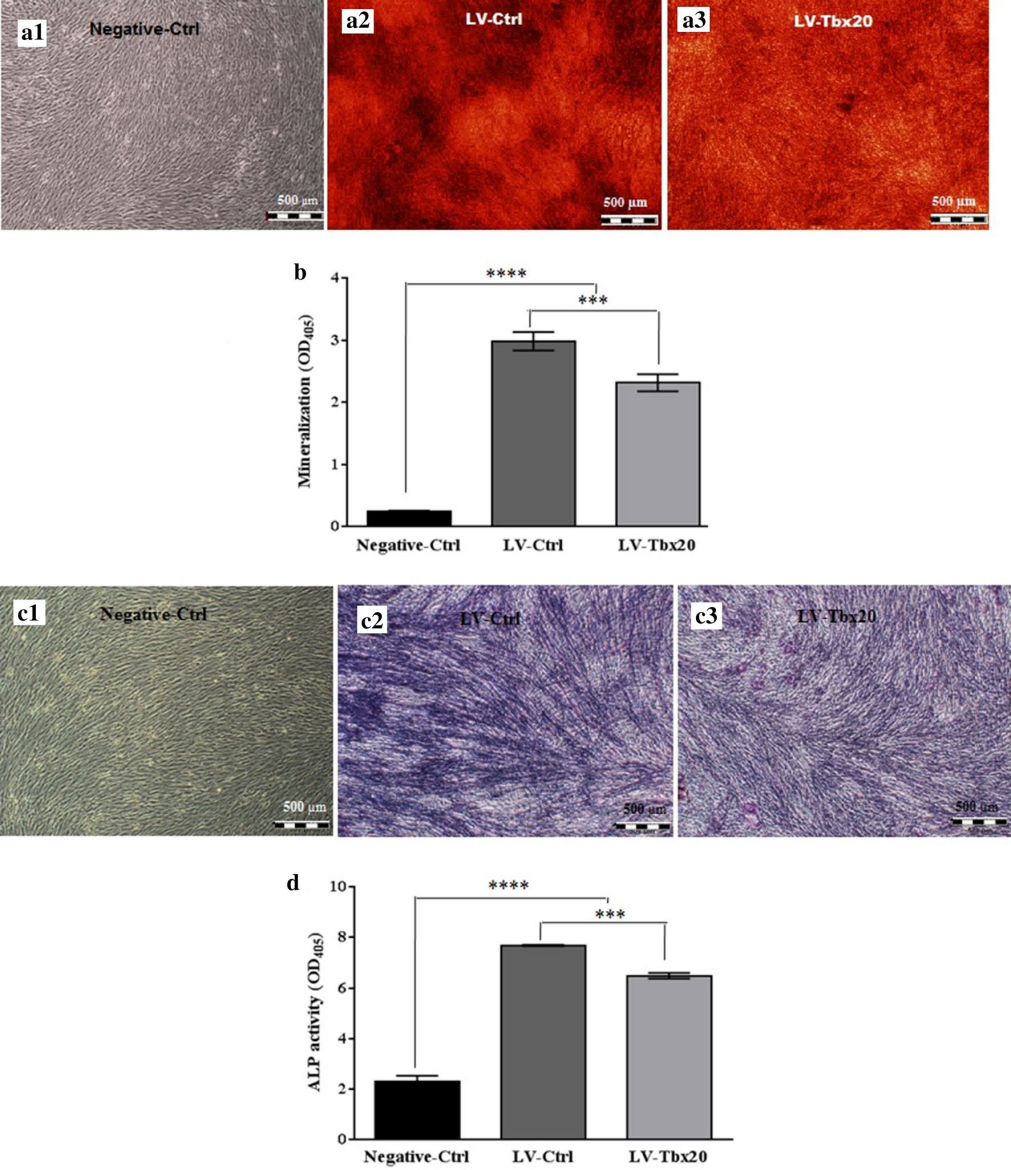
Enhanced expression of *Tbx20* in Ad-MSCs reduced osteogenesis. Cells were infected with control viral vector (LV-Ctrl) or LV-*Tbx20* and allowed to differentiate in osteogenic medium. **a** After 21 days, mineral nodules deposition was stained with Alizarin Red S (ARS). **b** The stained cells were extracted using 10% acetic acid and its absorbance was read at 405 nm to quantify the mineralization level. **c** After 21 days, induced cells were stained with BCIP/NBT to detect secreted alkaline phosphatase. **d** After 7 and 14 days ALP activity was conducted using pNPP liquid substrate system. All data are presented as mean ± SD. ****Shows significant differences (*p *< 0.0001) between negative control (cells grown in normal medium) and infected groups; ***Indicates significant differences (*p *< 0.001) between LV-Ctrl and LV-*Tbx20*. Experiments were performed in triplicate and repeated three times

**Fig. 4:**
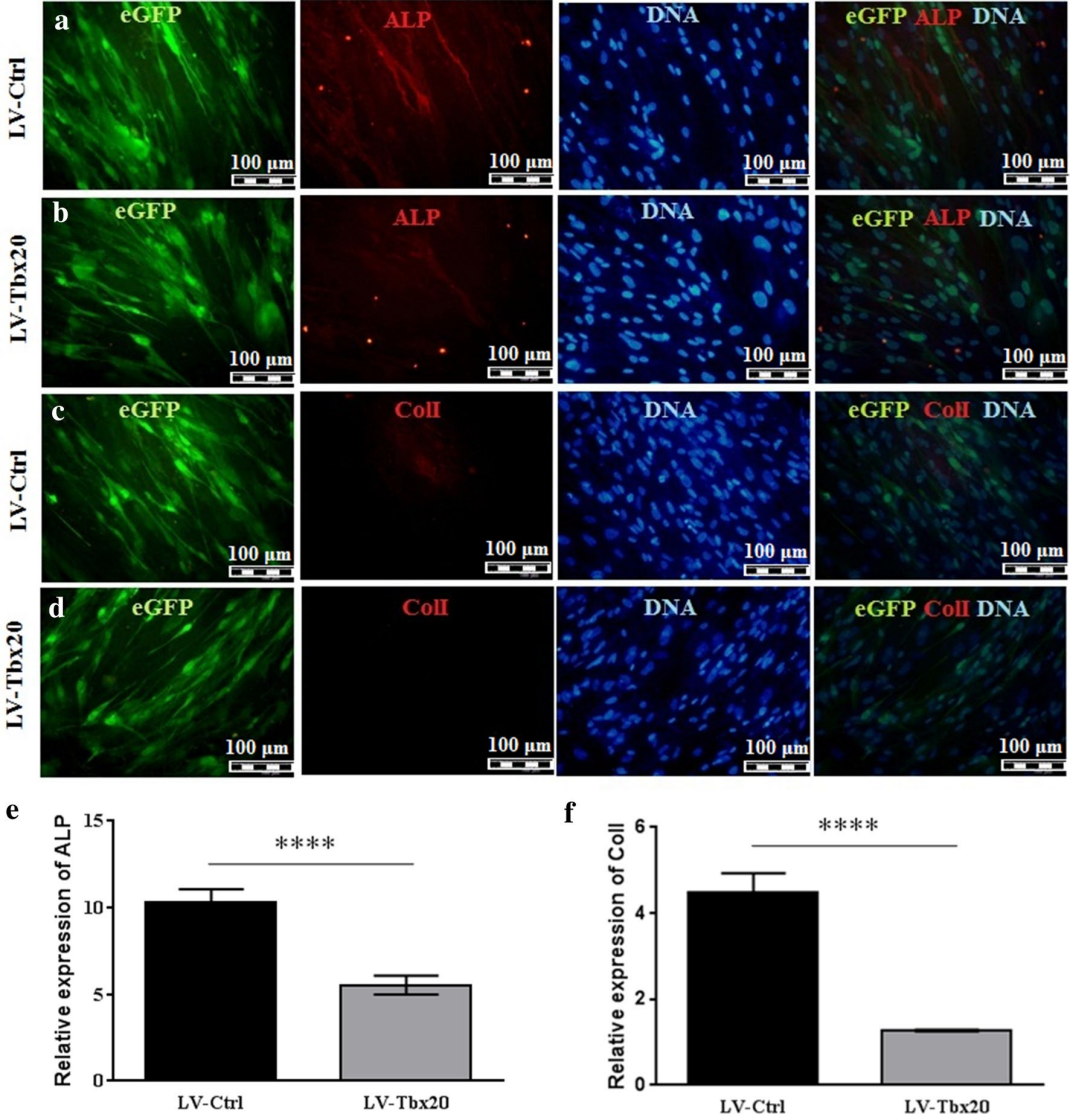
Increased expression of *Tbx20* decreased ALP and ColI expression. Representative micrograph of 14 days incubation of Ad-MSCs exposed to LV-Ctrl or LV-*Tbx20* with EGFP-positive, ALP and ColI expressions (**a**–**d**). Ad-MSCs overexpressed *Tbx20* demonstrated two- and threefolds reduction in ALP and ColI expression respectively compared to LV-Ctrl (**e**, **f**). ****Shows significant differences (*p *< 0.0001) between LV-Ctrl and LV-*Tbx20*. Experiments were performed in triplicate and repeated three times

To investigate the effects of *Tbx20* expression on osteogenesis, Ad-MSCs were transduced with LV-*Tbx20*, which expresses both *Tbx20* and EGFP, or with LV-Ctrl, which only codes for EGFP. The transduction efficiency of the Ad-MSCs at 72 h post-transduction was about 90% as judged by direct EGFP fluorescence microscopy (Additional file 2: Fig. S2A, B). The transduced cells were subsequently cultured in osteogenic differentiation medium for up to 21 days (Fig. 2a, b), after which they were subjected to ARS staining and ALP activity measurement. Besides, the expression of murine *Tbx20* after 14 days showed 20 times more expression in LV-*Tbx20* transduced cells in comparison with LV-Ctrl cells (Fig. 2c). The ARS staining (Fig. 3a) revealed that *Tbx20* overexpression reduced the formation of mineralized nodules in Ad-MSCs. Consistent with these findings, ARS quantification confirmed less calcification in LV-*Tbx20*-treated cells than in LV-Ctrl-transduced Ad-MSCs (Fig. 3b). Also, staining the cells with BCIP/NBT revealed that in LV-*Tbx20* group the blue cells were less prominent than LV-Ctrl group (Fig. 3c). ALP activity in *Tbx20*-overexpressing Ad-MSCs at 14 days after initiating osteogenesis was about 1.2-fold lower than in LV-Ctrl-transduced Ad-MSCs (Fig. 3d). This was accompanied by a reduction in immunostaining of ALP and ColI in LV-*Tbx20*-expressing cells in comparison with LV-Ctrl-transduced Ad-MSCs (Fig. 4). Collectively, these results demonstrate an inhibitory effects of *Tbx20* overexpression on the osteogenic differentiation ability of Ad-MSCs.

### The effect of *Tbx20* overexpression on osteoblastic gene expression in Ad-MSCs

**Fig. 5:**
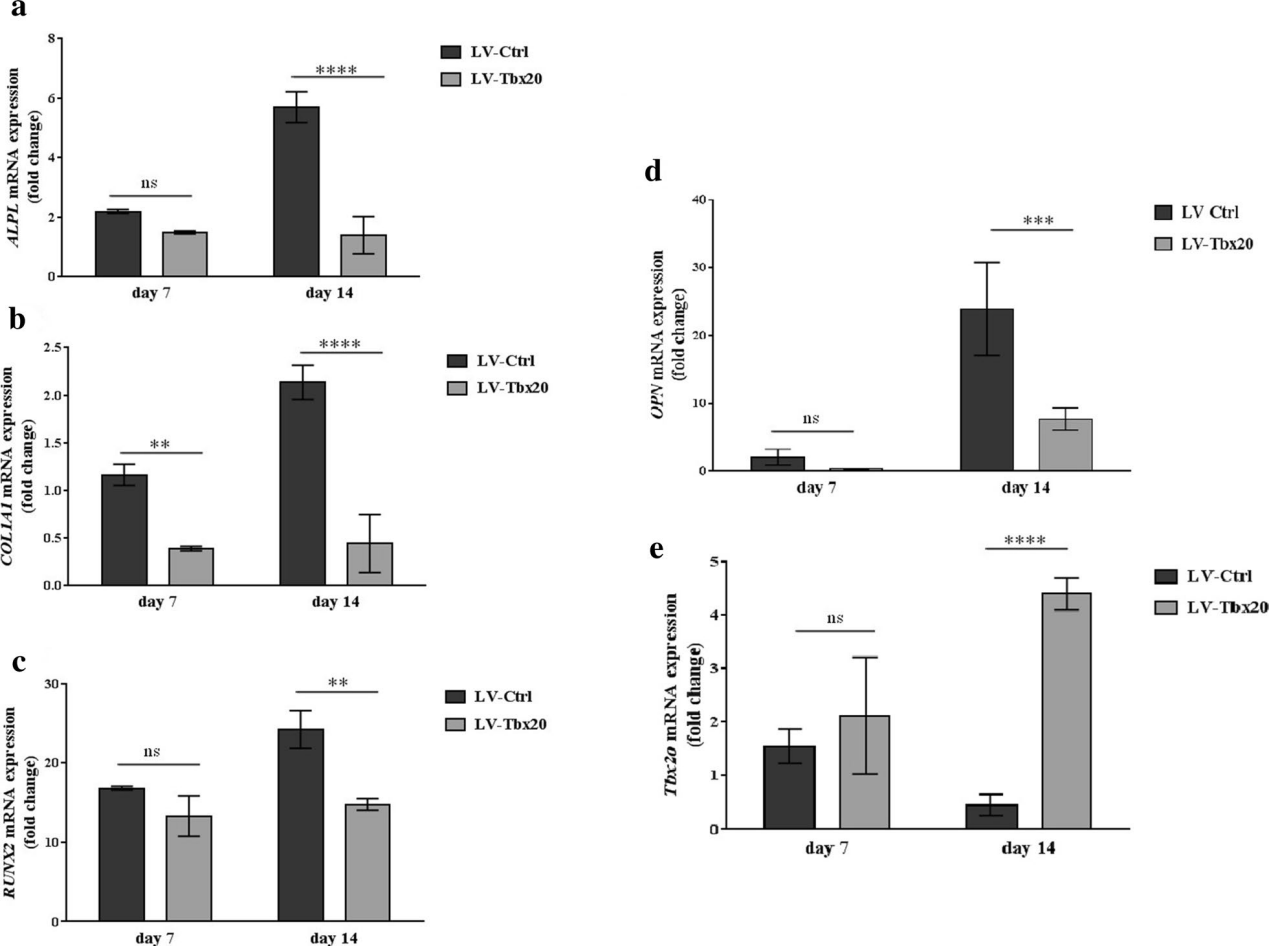
Analysis, by RT-qPCR, of the expression of the osteoblast marker genes *ALPL* (**a**), *COL1A1* (**b**), *RUNX2* (**c**), *OPN* (**d**), and human *Tbx20* (**e**) in LV-Ctrl- and LV-Tbx20-transduced Ad-MSCs that were maintained in osteogenic differentiation medium for 7 or 14 days. Expression levels were normalized to those in untreated cells (i.e. cells cultured in normal growth medium) as mean ± SD. The *GAPDH* gene served as internal control. ****p< 0.0001. ***p< 0.001. **p< 0.01. *ns* not significant. Experiments were performed in triplicate and repeated three times

To study the negative effects of *Tbx20* overexpression on osteogenesis in more detail, the mRNA levels of osteoblast-related genes in LV-*Tbx20*- and LV-Ctrl-transduced Ad-MSCs at different times after osteoinduction were evaluated by RT-qPCR. Overexpression of *Tbx20* reduced the expression of the osteoblast differentiation markers *ALPL* (Fig. 5a), *COL1A1* (Fig. 5b), *RUNX2* (Fig. 5c), and *OPN* (Fig. 5d) in Ad-MSCs in a time-dependent manner. In comparison, human *Tbx20* expression has been demonstrated in Fig. 5e. At 14 days after the initiation of osteogenesis, *ALPL*, *COL1A1*, *RUNX2*, and *OPN* mRNA levels were respectively, 4-, 4.6-, 1.6- and 3-fold lower in LV-*Tbx20*-treated Ad-MSCs than in LV-Ctrl-transduced Ad-MSCs (*p* < 0.05).

## Discussion

Tissue development is spatiotemporally regulated by transcription factors and noncoding RNAs including microRNAs, which either stimulate or inhibit the expression of target genes in a cell type-specific manner [25]. Several factors negatively regulate bone formation including microRNAs such as miR-378, miR-138, and miR-182 [26, 27] and transcription factors such as *NFE2* [2], *GATA4* [28], and *HOXC8* [29] as well as *p53* as a tumor suppressor [30]. Previously, we have demonstrated that *Tbx20* has cardiogenic effects [22]. In the present study, we introduced *Tbx20* in Ad-MSCs using a HIV1-based lentiviral vector to test its modulatory effects on osteogenesis. Our results demonstrated that *Tbx20* reduced the osteoblastic differentiation of Ad-MSCs. ARS staining revealed decreased calcified nodule formation and ALP assays showed a reduction in ALP activity in *Tbx20*-overexpressed cells compared to control cells. Moreover, the proteins expression of ALP and ColI significantly decreased in LV-Tbx20-treated Ad-MSCs in comparison to LV-Ctrl-transduced Ad-MSCs. Similar results were obtained by RT-qPCR in which it has been demonstrated that *Tbx20* overexpression significantly down-regulated *ALPL*, *COL1A1*, *RUNX2*, and *OPN* expressions on Ad-MSCs in a time dependent manner. Possible mechanisms involved in the osteosuppressive effects of *Tbx20* could be explained by its binding to important osteoblast transcription factors and changing their function and/or expression at key stages of differentiation. It has been well-documented that *Tbx20* can act as a transcriptional repressor [31], raising the possibility that *Tbx20* could down-regulate osteoblastic gene expression in Ad-MSCs. *RUNX2* is a key transcription factor regulating expression of various osteoblast marker genes including those encoding type Iα1 collagen, osteocalcin, ALP, and osteopontin [32]. As it was assumed, overexpression of *Tbx20* in Ad-MSCs indeed down-regulated *RUNX2*, *ALPL* and *COL1A1* mRNA levels. It has been reported that *Tbx3* blocks osteoblast differentiation through inhibition of *Osterix* and *RUNX2* [4]. As mentioned, the members of T-box family have similar DNA-binding properties [10, 11]. Therefore, a more likely explanation for our findings could be that *Tbx20* similar to *Tbx3* regulated osteogenesis by binding to master transcription factor i.e. *RUNX2*. Consistently, it was shown that increased expression of *Tbx3* could mislocalize *RUNX2* [33]. Similarly, *Tbx15* deficiency in mutant mice not only leads to reduction in bone size and length but also delays endochondral bone formation. Interestingly, overexpression of *Tbx2* in NIH3T3 cell line up-regulates osteoblastogenesis and chondroblastogenesis [34]. Accordingly, we could not ignore the potential role of *Tbx20* in regulation of bone formation although some probable functions of recombinant *Tbx20* could be hindered by spatial interference with EGFP in our experiments. *Tbx20* had been reported to transcriptionally activate peroxisome proliferator-activated receptor gamma (*PPAR*-*γ*) gene expression to protect endothelial cells against oxidative stress [31] and to regulate energy pathways [35]. The balance between osteogenesis and adipogenesis is regulated via two master transcription factors namely *PPAR*-*γ* and *RUNX2* [36]. Since *PPAR*-*γ* have an anti-osteoblastogenic effects, its activation inhibits osteogenesis and promotes adipogenesis [36]. So, another explanation for our findings could be the activation of *PPAR*-*γ* by *Tbx20*. The study by Brugmann et al. [17] has indicated a dual role of *Tbx20* in modulating of the Wnt signaling pathway. It was demonstrated that *Tbx20* regulates Wnt signaling through direct binding to the *Lef1’*s promoter [37]. Particularly, Wnt signaling has been involved in the osteogenic differentiation of mesenchymal stem cells [38, 39]. Therefore, our data could be possibly explained by an inhibitory role of *Tbx20* on *RUNX2* expression or through repression of the Wnt signaling pathway in *Tbx20*-overexpressing Ad-MSCs. Negative osteogenesis regulation of *Tbx20* in Ad-MSCs may indicate that *Tbx20* could play a role in bone diseases. Further studies are needed to identify key binding partners for *Tbx20* in osteoblasts.

## Conclusion

*Tbx20* could inhibit osteoblast differentiation during critical stages of osteogenesis probably through binding to *PPAR*-*γ* or repressing Wnt signaling followed by reduction of osteoblastic gene expression. This study provides a rationale for further investigating the role of *Tbx20* in osteoblast differentiation as well as in postnatal bone formation in an attempt to develop new treatment modalities for bone-related diseases.

## Supplementary information

**Additional file 1: Figure S1.** Adipogenic and osteogenic differentiation of human Ad-MSCs. Cells were grown for 21 days in adipogenic and osteogenic differentiation media. Fat vacuoles and mineralization were visualized by Oil Red O (A) and Alizarin Red S stainings (B), respectively.

**Additional file 2: Figure S2.** Fluoromicrographs of human Ad-MSCs transduced with LV-Ctrl (A) or LV-*Tbx20* (B). The pictures were taken at 3 days post transduction.

## Data Availability

All other data is available from the corresponding author upon request.

## Abbreviations

Ad-MSCs: adipose-derived human mesenchymal stem cells
HIV1: human immunodeficiency virus type 1
DMEM-LG: Dulbecco’s modified Eagle’s medium-low glucose
FBS: fetal bovine serum
EGFP: enhanced green fluorescent protein
HEK: human embryonic kidney
PEI: polyethylenimine
ARS: Alizarin Red S
pNPP: *p*-nitro-phenyl phosphate
ALP: alkaline phosphatase
ColI: collagen type I
*GAPDH*: glyceraldehyde 3-phosphate dehydrogenase
OPN: osteopontin
RT: room temperature
