# Supplementary figures and images for: T-Box20 inhibits osteogenic differentiation in adipose-derived human mesenchymal stem cells: the role of T-Box20 on osteogenesis
Source: J Biol Res (Thessalon). 2019 Sep 18;26:8. doi: 10.1186/s40709-019-0099-5 (PMC6751895; doi:10.1186/s40709-019-0099-5)

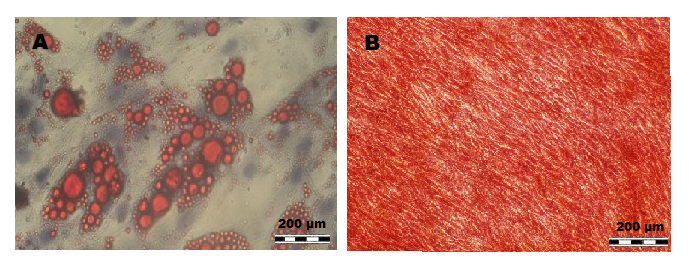

Supplement: Supplementary file 1 — Additional file 1: Figure S1. Adipogenic and osteogenic differentiation of human Ad-MSCs. Cells were grown for 21 days in adipogenic and osteogenic differentiation media. Fat vacuoles and mineralization were visualized by Oil Red O (A) and Alizarin Red S stainings (B), respectively. [file 40709_2019_99_MOESM1_ESM.tif]

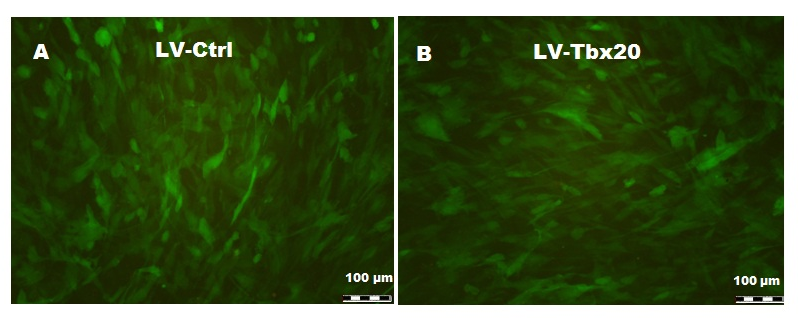

Supplement: Supplementary file 2 — Additional file 2: Figure S2. Fluoromicrographs of human Ad-MSCs transduced with LV-Ctrl (A) or LV-Tbx20 (B). The pictures were taken at 3 days post transduction. [file 40709_2019_99_MOESM2_ESM.tif]
